# Calsequestrin Deletion Facilitates Hippocampal Synaptic Plasticity and Spatial Learning in Post-Natal Development

**DOI:** 10.3390/ijms21155473

**Published:** 2020-07-31

**Authors:** Patrizia Ambrogini, Davide Lattanzi, Michael Di Palma, Caterina Ciacci, David Savelli, Claudia Galati, Anna Maria Gioacchini, Laura Pietrangelo, Luciana Vallorani, Feliciano Protasi, Riccardo Cuppini

**Affiliations:** 1 Department of Biomolecular Sciences, Università di Urbino Carlo Bo, I-61029 Urbino, Italy; davide.lattanzi@uniurb.it (D.L.); caterina.ciacci@uniurb.it (C.C.); david.savelli@uniurb.it (D.S.); caludia.galati@uniurb.it (C.G.); anna.gioacchini@uniurb.it (A.M.G.); luciana.vallorani@uniurb.it (L.V.); riccardo.cuppini@uniurb.it (R.C.); 2 Department of Experimental and Clinical Medicine, Faculty of Medicine and Surgery, Università Politecnica delle Marche, I-60121 Ancona, Italy; m.dipalma@univpm.it; 3 Center for Advanced Studies and Technology & Department of Medicine and Aging Sciences, Università G. d’Annunzio, I-66100 Chieti, Italy; laura.pietrangelo@unich.it (L.P.); feliciano.protasi@unich.it (F.P.)

**Keywords:** calcium dynamics, cognitive functions, neural plasticity, hippocampus, pyramidal neurons, adult mouse

## Abstract

Experimental evidence highlights the involvement of the endoplasmic reticulum (ER)-mediated Ca^2+^ signals in modulating synaptic plasticity and spatial memory formation in the hippocampus. Ca^2+^ release from the ER mainly occurs through two classes of Ca^2+^ channels, inositol 1,4,5-trisphosphate receptors (InsP3Rs) and ryanodine receptors (RyRs). Calsequestrin (CASQ) and calreticulin (CR) are the most abundant Ca^2+^-binding proteins allowing ER Ca^2+^ storage. The hippocampus is one of the brain regions expressing CASQ, but its role in neuronal activity, plasticity, and the learning processes is poorly investigated. Here, we used knockout mice lacking both CASQ type-1 and type-2 isoforms (double (d)CASQ-null mice) to: a) evaluate in adulthood the neuronal electrophysiological properties and synaptic plasticity in the hippocampal Cornu Ammonis 1 (CA1) field and b) study the performance of knockout mice in spatial learning tasks. The ablation of CASQ increased the CA1 neuron excitability and improved the long-term potentiation (LTP) maintenance. Consistently, (d)CASQ-null mice performed significantly better than controls in the Morris Water Maze task, needing a shorter time to develop a spatial preference for the goal. The Ca^2+^ handling analysis in CA1 pyramidal cells showed a decrement of Ca^2+^ transient amplitude in (d)CASQ-null mouse neurons, which is consistent with a decrease in afterhyperpolarization improving LTP. Altogether, our findings suggest that CASQ deletion affects activity-dependent ER Ca^2+^ release, thus facilitating synaptic plasticity and spatial learning in post-natal development.

## 1. Introduction

Calcium ions (Ca^2+^) play a crucial role as second messengers in all cell types [1]. Transient elevations of intracellular Ca^2+^ concentration ([Ca^2+^]_i_) are pivotal for many cellular functions [2]. In neurons, the rise of cytosolic Ca^2+^ concentration triggers the release of neurotransmitter at the synaptic junction, contributes to action potential, and regulates the activity-dependent changes in gene expression [3,4]. Being so versatile, Ca^2+^ signals are also critical for cellular and molecular mechanisms underlying synaptic plasticity [4,5,6], crucial in physiological brain functions, such as learning and memory [7]. Neuronal Ca^2+^ signals arise either from Ca^2+^ entry, mostly via voltage-gated or receptor-operated Ca^2+^ channels; via store-operated Ca^2+^ entry (SOCE) [8]; and/or from Ca^2+^ release from intracellular stores (i.e., endoplasmic reticulum, ER).

The ER represents the main endogenous reservoir of Ca^2+^ in all cell types, including neurons, where it extends as a continuous endomembrane structure from the cell soma toward dendrites and dendritic spines, as well as axons and pre-synaptic terminals [9]. Ca^2+^ release from the ER may occur via the inositol 1,4,5-trisphosphate receptor (InsP3R) or the ryanodine receptor (RyR) Ca^2+^ channels. InsP3R and RYR are two large homotetrameric proteins (~1.2 and ~2 MDa, respectively), and represent the major intracellular Ca^2+^ release channels of the ER. Ca^2+^ release from InsP3Rs and RyRs produces intracellular Ca^2+^ signals [10] that make a significant contribution to synaptic plasticity processes in central neurons [11]. In particular, RyR-mediated Ca^2+^ release, which is controlled by a Ca^2+^-induced Ca^2+^ release (CICR) mechanism [9,10], provides a preeminent fraction of the increase in intracellular Ca^2+^ induced in hippocampal Cornu Ammonis 1 (CA1) spines by synaptic activity [12]. In keeping with these observations, recent experimental evidence highlights the implications of ER-mediated Ca^2+^ signals in modulating the duration, extent, and type of synaptic plasticity [13] and the formation/consolidation of spatial memory in the hippocampus [14].

The large amount of Ca^2+^ stored in the ER creates the driving force required for Ca^2+^ release, and regulates the functional availability of Ca^2+^ release channels. The capability of the ER to store Ca^2+^ depends on several low affinity Ca^2+^-binding proteins, which enable the maintenance of high intra-ER free Ca^2+^ levels [15]. In neurons, calsequestrin (CASQ) and calreticulin (CR) are the two most abundant Ca^2+^-binding proteins, which may bind up to 50 Ca^2+^ ions with a low affinity. The two proteins exhibit a dramatic region-specific distribution in the brain, with CR expression low in the hippocampus, while CASQ is mostly expressed in the hippocampus [16].

CASQ and CR interact, respectively, with RyRs and InsP3Rs to regulate Ca^2+^ release from the ER during cell rest and activity. In particular, CASQ is well known to control RyR channel activity in muscles, where it is proposed to reduce the RyR opening probability, stabilizing the closing state of the channel. In striated muscles, it is expressed in two isoforms, which are products of two different genes, CASQ1 and CASQ2 [17,18]. CASQ2 is the only isoform expressed in the heart independently from the developmental stage, whereas both CASQ genes are transcribed in skeletal muscles at different rates in slow- and fast-twitch fibers [19,20]. The ablation of CASQ1 in skeletal muscle has been associated with structural and functional changes in muscle fibers [21,22] and susceptibility to developing hyperthermic crises in response to the administration of anesthetics, heat, and exercise [23,24,25,26]. Ablation or mutations in CASQ2 have been linked to a rare form of tachycardia in children and mice [27,28], whereas the first mutations in the CASQ1 gene have been identified in families affected by vacuolar myopathy [29,30].

Although it has been demonstrated that CASQ is expressed in the hippocampus [16], very little is known about its role in neuronal activity and synaptic plasticity. This role may be hypothesized considering the involvement of intra-store Ca^2+^ binding proteins in the modulation of [Ca^2+^]_i_ transients [15] and the implications of the latter in synaptic plasticity [31,32]. Thus, to gain insight about this topic, we used CASQ knockout mice lacking both CASQ1 and CASQ2 isoforms, double (d)CASQ-null mice, generated by cross-breeding the preexisting CASQ1-null and CASQ2-null mice [21,22,27]. In these mice, we evaluated the electrophysiological properties of pyramidal neurons in the CA1 field of the hippocampus, including Ca^2+^ imaging, and synaptic plasticity by long-term potentiation (LTP) induction. Moreover, the performances of mice in spatial learning tasks were also investigated using the paradigm of the Morris Water Maze. We mainly found that CASQ2 is the isoform expressed in the hippocampus, and that the lack of this protein results in synaptic plasticity enhancement and spatial learning improvement, possibly by affecting the Ca^2+^ dynamics. To our knowledge, this is the first work that provides insights about the involvement of CASQ in hippocampal neuron structure and function.

## 2. Results

Mice knockout for both CASQ isoforms, (d)CASQ-null mice, are viable and fertile [22]. (d)CASQ-null mice develop and breed normally, and do not show behavioral alteration under standard housing conditions. However, (d)CASQ-null male mice exhibit an increased mortality rate compared to control (CTRL) animals [22,23,24].

### 2.1. Biochemical Measurements

**Figure 1:**
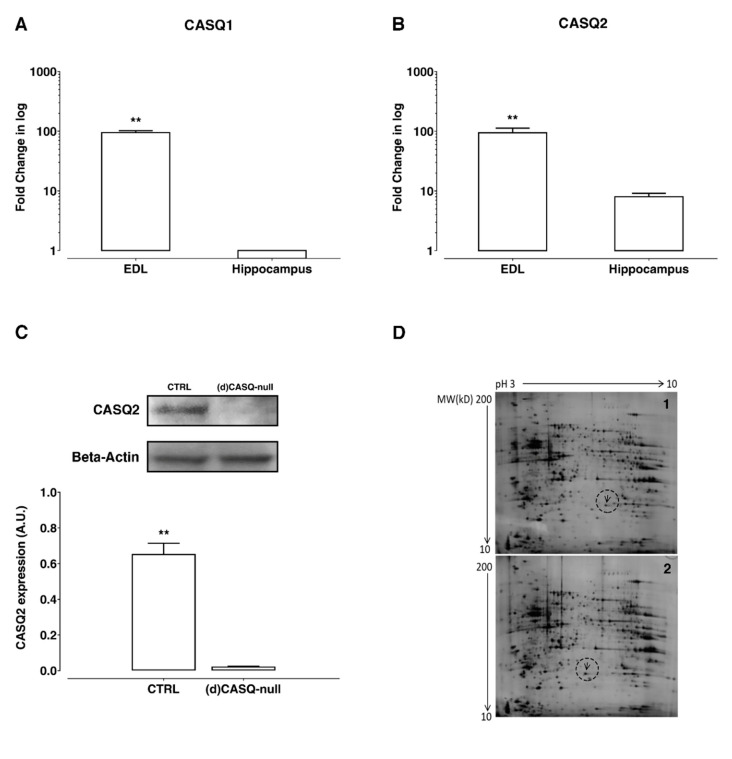
Biochemical evaluations in hippocampi. (**A**,**B**) Calsequestrin (CASQ) 1 and CASQ2 mRNA levels in the hippocampus of control (CTRL) mice vs. extensor digitorum longus (EDL) muscle of the same group (*n* = 3). Student’s t-test: CASQ1 t(22) = 20.95, ** *p* < 0.01; CASQ2 t(22) = 7.01_,_ ** *p* < 0.01. (**C**) CASQ2 protein expression in the hippocampus of CTRL vs. (d)CASQ-null mice (*n* = 3 for each group). Bars represent the signal intensity of protein bands in arbitrary units (A.U.) after normalization with the signal intensity of Beta-Actin used as a loading control for each sample. Student’s t-test: t(16) = 10.26, ** *p* < 0.01. (**D**) Two-dimensional gel electrophoresis (2DE) maps of hippocampus proteins from CTRL (1) and (d)CASQ-null mice (2) (*n* = 3 for each group). Arrows point to differentially expressed proteins.

The expression of CASQ is well documented in mammalian skeletal and cardiac muscle [17,18,19,20]. On the other hand, the insight about CASQ isoform expression in the hippocampus is still very feeble. Therefore, the transcripts of both CASQ isoforms were analyzed in the hippocampus of CTRL mice. The quantification of CASQ1 and CASQ2 mRNA was carried out using extensor digitorum longus (EDL) skeletal muscle as reference tissue (Figure 1A,B). The data obtained indicate that in CTRL hippocampus, CASQ2 mRNA was the predominant isoform (Figure 1A,B), being the appearance of CASQ1 mRNA near cut-off cycles.

The expression of the two CASQ isoforms in CTRL and (d)CASQ-null mice was assessed by Western blot (WB) in homogenates prepared from hippocampi. CASQ2 was the only isoform detected by WB in CTRL mouse hippocampi, in line with the mRNA expression data (Figure 1C). No expression of CASQ2 was obtained in the hippocampi of (d)CASQ-null mice (Figure 1C).

**Figure 2:**
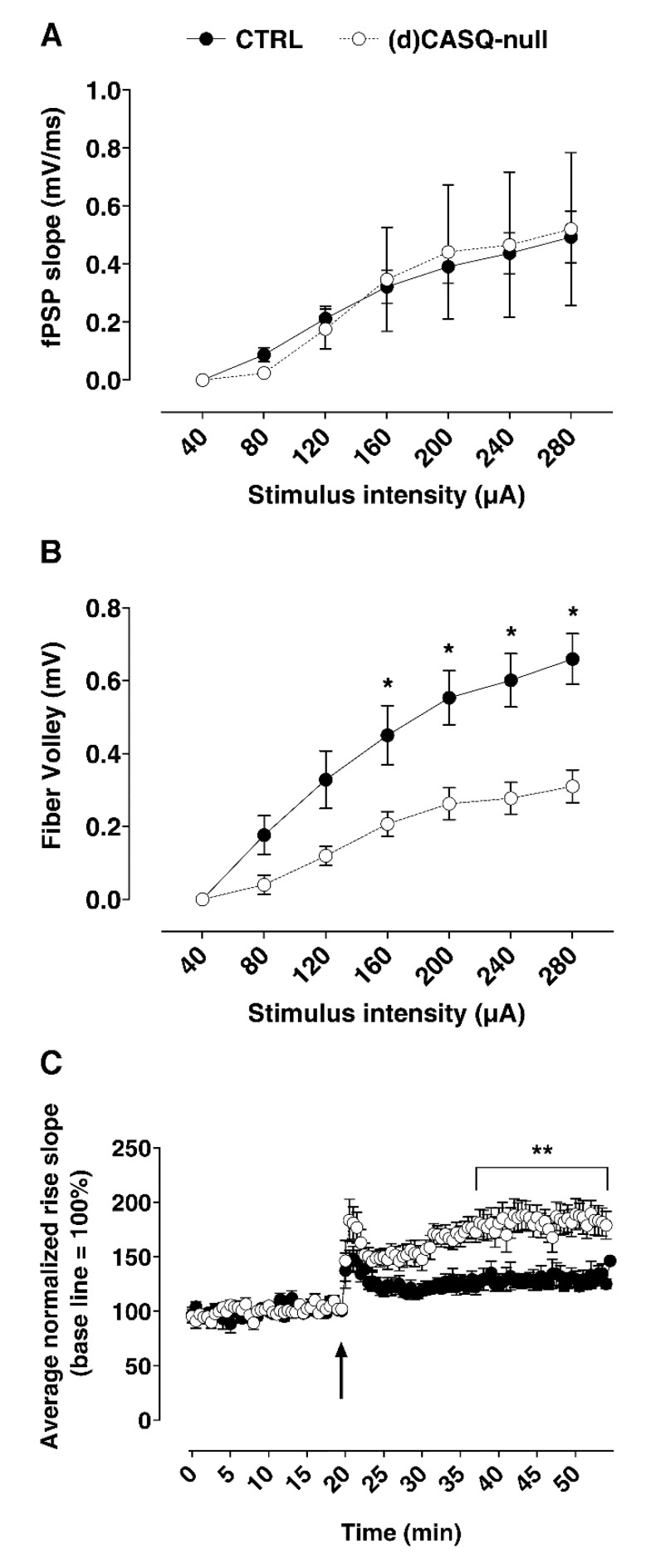
Extracellular field post-synaptic potentials (fEPSPs) recorded in the CA1 stratum radiatum. (**A**) Relationship between the fEPSP slope and the stimulus intensity. (**B**) Fiber volley amplitude vs. stimulus intensity. CTRL (*n* = 8 slices from 6 animals) vs. (d)CASQ-null (*n* = 6 slices from 5 animals). Two-Way ANOVA test: F(1, 84) = 49.42, *p* < 0.01; Sidak’s multiple comparisons test: * *p* < 0.05, ** *p* < 0.01. (**C**) fEPSP slope (10% and 80% of max) recorded before and after the Schaffer Collateral high-frequency stimulation: the arrow indicates the stimulus application. Values were normalized to the mean value obtained over the last 20 min of the baseline period and expressed as a percentage of this baseline value (*n* = 9 slices from 6 animals vs. *n* = 10 slices from 5 animals in CTRL and (d)CASQ-null, respectively). Two-way ANOVA test: F(1,17) = 13.06, *p* < 0.01; Sidak’s multiple comparisons test: ** *p* < 0.01.

To gain insight into protein expression in CASQ deletion conditions, a proteomic analysis was also performed. A total of 830 spots (mean) were detected on each analytical gel obtained by 2-DE of the proteins isolated from hippocampi of (d)CASQ-null and CTRL mice. As shown in Figure 1D, the comparison between the 2-DE maps revealed no significant differences in protein expression except for the proteins marked with arrows, which were present in the hippocampi of (d)CASQ-null mice (Figure 1D-1, arrow) and CTRL mice (Figure 1D-2, arrow), respectively. The two differentially expressed proteins showed a very similar molecular weight, but a different isoelectric point value. The protein in CTRL had an isoelectric point of about 6.0, while that in the (d)CASQ-null was more acidic (pI 5.7). Electrospray ionization (ESI) quadrupole time-of-flight (QTOF) mass spectrometry revealed that, in both cases, the protein was the peroxiredoxin 6 with a difference found in its the primary structure—i.e., the amino acid residue in position 124 was alanine in CTRL and aspartic acid in (d)CASQ-null mice.

### 2.2. Electrophysiological Analysis

#### 2.2.1. Field Synaptic Responses

Field recordings were used to investigate synaptic plasticity in the hippocampus of mutant and CTRL mice by evaluating their basal synaptic transmission (input/output curves) and ability to elicit LTP.

Basal synaptic transmission in the Schaffer collateral-CA1 pathway was investigated by input/output curves in CTRL and (d)CASQ-null mice. The field potential responses to increasing intensity stimuli of the Schaffer collaterals were not significantly different in the two groups of samples (Figure 2A). On the other hand, the relationship between the fiber volley amplitude and the stimulus intensity showed that a given intensity stimulus activated a lower number of fibers in the (d)CASQ-null group (Figure 2B). Thus, to elicit a field postsynaptic response similar to that of CTRLs, a smaller number of presynaptic terminals would be recruited in mutant mice compared to controls.

(d)CASQ knockout was also found to affect the LTP ability. In CTRL slices, high-frequency stimulations (HFS) of Schaffer collaterals induced, in CA1 pyramidal neurons, a potentiation of the field post-synaptic potentials (fEPSP) response (LTP), which, after an initial decrease, remained stable at least up to 40 min following LTP induction (Figure 2C). In slices from (d)CASQ-null mouse hippocampi, the induced LTP was similar to that of controls over the first minutes post-HFS, but the fEPSP decrement was much less pronounced than in CTRLs. Moreover, the LTP stabilized at significantly higher values compared to the controls, and did not vary at least up to 40 min post-HFS (Figure 2C). Therefore, CASQ deletion may enhance synaptic plasticity, inducing an improvement in LTP maintenance.

#### 2.2.2. Whole-Cell Patch Clamp

To investigate the functional features of CA1 hippocampal neurons under CASQ deletion conditions for providing an explanation of results obtained by field recordings, the whole-cell patch clamp technique was used and the electrophysiological properties of (d)CASQ-null CA1 neurons compared to CTRLs were analyzed. The membrane passive properties (IR and C) of CA1 pyramidal cells were not different between the (d)CASQ-null and CTRL groups (Table 1), whereas the resting membrane potential (RMP) resulted in being significantly depolarized in (d)CASQ-null mice compared to the CTRLs. Likewise, even if the action potential amplitude was similar between the two groups of samples, the AP threshold was significantly less depolarized in the (d)CASQ-null mice than the CTRLs and, thus, closer to the CA1 pyramidal cell RMP (Table 1). Thus, CASQ-null conditions would induce an increased excitability.

**Figure 3:**
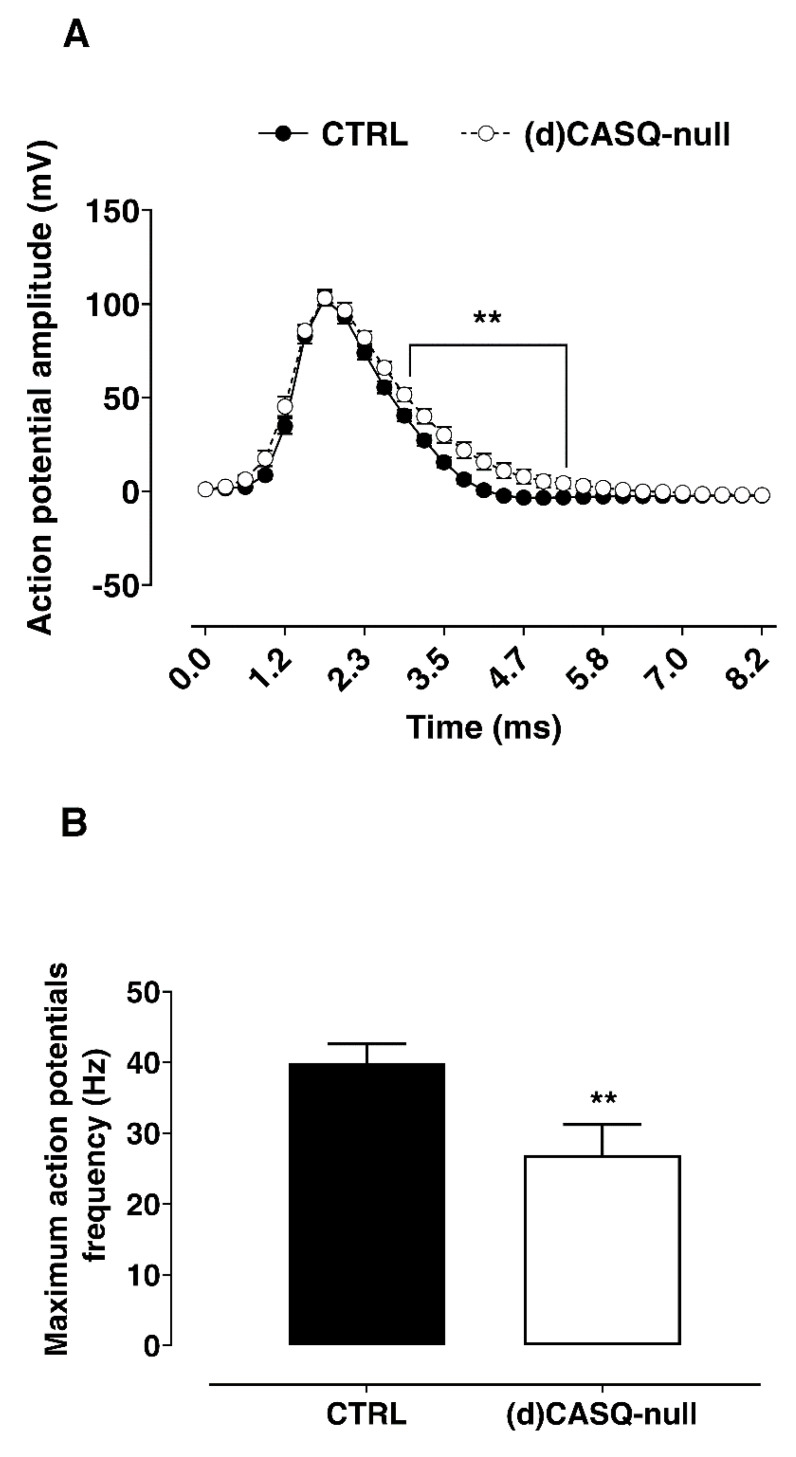
CA1 pyramidal neuron excitability. (**A**) Averaged action potentials recorded in CTRL (filled circle; *n* = 23 cells from 6 animals) and in (d)CASQ-null mice (open circles; *n* = 19 cells from 6 animals) aligned horizontally by peak and vertically by threshold. Two-way ANOVA test: F(1, 40) = 8.04, *p* < 0.01; Sidak’s multiple comparisons test ** *p* < 0.01. (**B**) Maximum action potential frequency reached in CTRL (*n* = 23 cells from 6 animals) and (d)CASQ-null mice (*n* = 18 cells from 6 animals). Student’s t-test: t(40) = 2.65, ** *p* < 0.01.

In addition, the analysis of the average action potential in CA1 neurons of (d)CASQ-null mice showed a significantly slower repolarizing phase and the lack of the hyperpolarizing phase in comparison to the corresponding CTRL (Figure 3A). Consistently with the slow repolarizing phase of AP, we also recorded a significant lower maximum AP frequency in CA1 neurons from (d)CASQ-null compared to CTRL samples (Figure 3B). The peak amplitude of afterhyperpolarization (AHP), elicited after spike burst induction, was found to be significantly decreased in (d)CASQ-null mice compared to the CTRLs (respectively: 1.66 ± 0.50 mV, *n* = 16; 4.21 ± 0.75 mV, *n* = 19; unpaired Student’s t-test: *p* = 0.01). Since the AHP was found to be inversely related to the LTP induction facility [33], our result would support the improvement in LTP maintenance in (d)CASQ-null mice.

Finally, no difference was found in the evoked synaptic response (PSCs) recorded in the CA1 neurons of the two experimental groups (PSC amplitude: unpaired Student’s t-test: *p* = 0.81).

#### 2.2.3. Calcium Imaging

To gain a better understanding of the mechanisms underlying the observed effects, the intracellular Ca^2+^ dynamics (endogenous Ca^2+^ buffer capacity, resting Ca^2+^ concentration, Ca^2+^ influx) were examined using fluorescence-based Ca^2+^ imaging techniques. Fluo-4 was put in the CA1 neuron cytoplasm at various concentrations through the patch pipette intracellular solution, resulting in neurons containing different exogenous Ca^2+^ buffer capacities (κ_B_).

**Figure 4:**
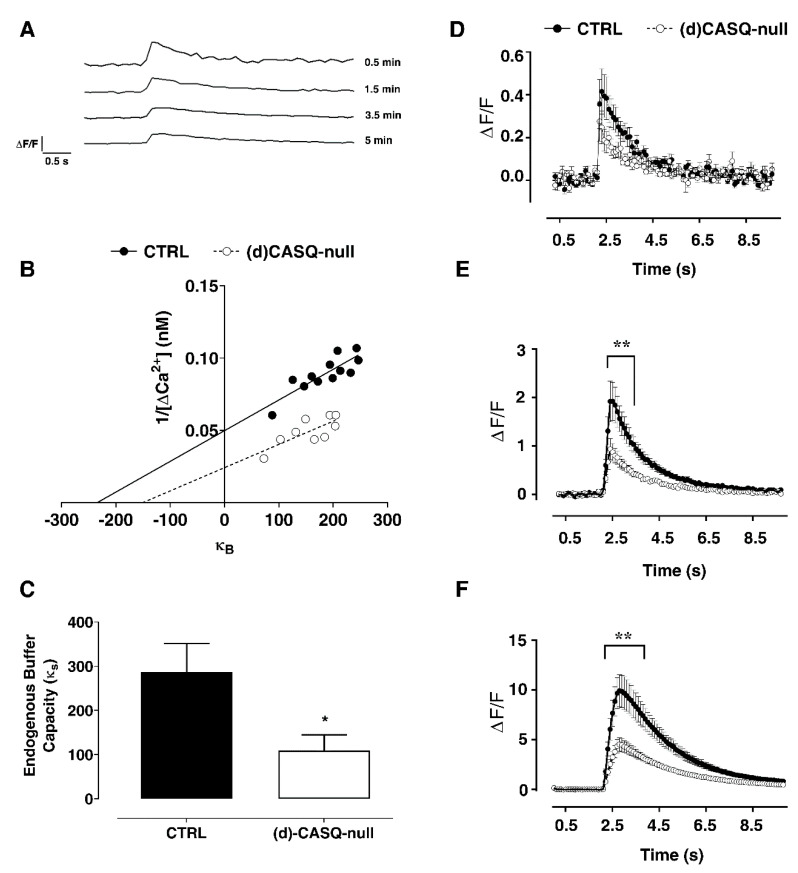
Calcium dynamics in CA1 pyramidal neurons. (**A**) Fluorescent transients elicited by single action potential at different time points after break-in (ΔF/F). (**B**) Examples of inverse single action potential peak transient (1/Δ[Ca^2+^]) vs. added buffer capacity (κ_B_) during the loading phase for a neuron from CTRL and (d)CASQ-null mice. (**C**) The endogenous Ca^2+^ buffer capacity (κ_S_) is shown. CTRL (*n* = 7 cells from 4 animals) vs. (d)CASQ-null (*n* = 6 cells from 3 animals). Student’s t-test: t(11) = 2.28, * *p* < 0.05. (**D**–**F**) Averaged (Ca^2+^) transient elicited by a single action potential and by the action potential trains of 20 Hz (E) and 100 Hz (F) recorded in CTRL (*n* = 11 cells from 4 animals) and (d)CASQ-null mice (*n* = 10 cells from 4 animals). Two-way ANOVA test: 20 Hz, F(1, 19) = 10.82 *p* < 0.01; Sidak’s multiple comparisons test ** *p* < 0.01 (from 2.3 s to 3.4 s); 80Hz, F(1, 18) = 10.57 *p* < 0.01; Sidak’s multiple comparisons test ** *p* < 0.01 (from 2.4 s to 4.3 s).

Consistent with the literature, we found that the baseline fluorescence intensity increased with the increasing concentration of intracellular fluorophore, reaching a steady state. The transients elicited by a single action potential were measured at different time points after break-in (ΔF/F), showing a transient amplitude decrease by increasing the added buffer capacity (Figure 4A).

The Ca^2+^ concentration at rest, [Ca^2+^]_0_, showed a trend towards higher values in (d)CASQ-null mice, which however was not found to be significantly different in the two experimental groups (Table 2). We then assessed the unperturbed changes in [Ca^2+^]_i_, evoked by a single action potential, that would have been detected in the absence of exogenous Ca^2+^ buffer (κ_B_; Table 2 and Figure 4B). Additionally, in this case the difference between the two experimental groups was not significant, even though a trend towards higher values could be noticed in (d)CASQ-null mice (Table 2, Figure 4B). Very similar was the Δ[Ca^2+^]_AP_ (i.e., [Ca^2+^]_i_ transient evoked by a single action potential) in (d)CASQ-null and CTRL mice (Table 2; Figure 4D) as well. However, the [Ca^2+^]_i_ transient amplitude decreased in the (d)CASQ-null mice in comparison with the CTRLs when elicited by action potential trains of 20–100 Hz (Figure 4E,F), suggesting less calcium influx and possibly calcium-induced calcium release under high-frequency stimulation in CASQ deletion conditions. Finally, the endogenous buffer capacity (κ_S_) was significantly reduced in conditions of CASQ deletion (Figure 4B: κ_B_: x-intercept—1; Figure 4C), indicating a lower ability of cells to handle Ca^2+^ load.

### 2.3. Morphological Analysis

**Figure 5:**
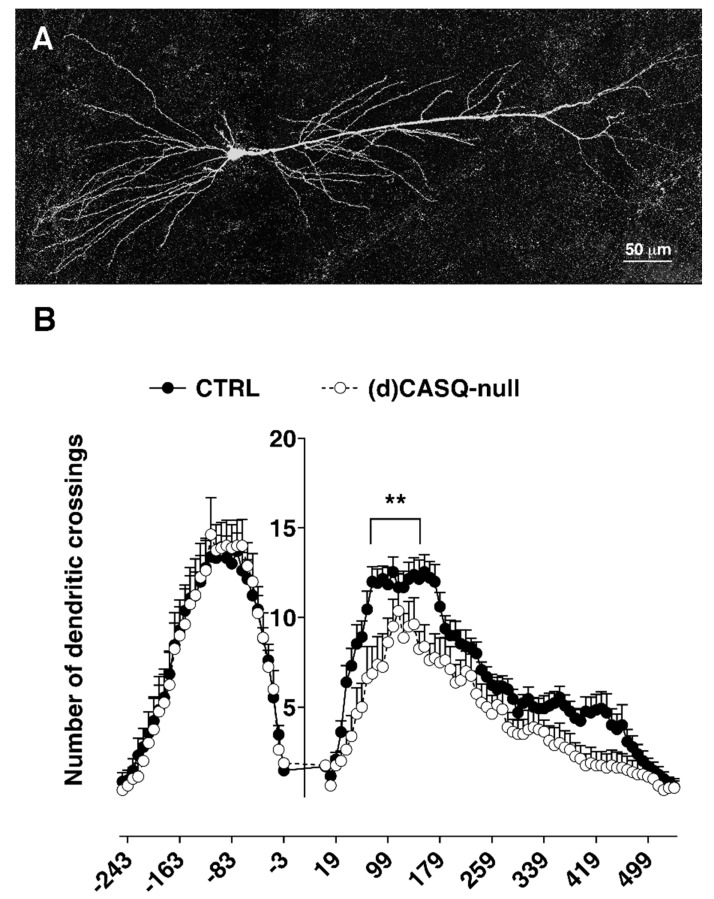
Dendritic tree complexity analysis of CA1 pyramidal neurons. (**A**) Picture showing an example of CA1 pyramidal neuron filled with biocytin and reconstructed by a confocal microscope used to perform the Sholl analysis. (**B**) The graph represents the number of dendritic crossings along the concentric Sholl rings at different distances from the soma. *n* = 13 cells from 6 animals vs. *n* = 8 cells from 6 animals in CTRL and (d)CASQ-null, respectively. Two-way ANOVA test: apical dendrites F(1, 19) = 7.196, *p* < 0.05; Sidak’s multiple comparisons test (75 µm ** *p* < 0.01; 83 µm ** *p* < 0.01; 91 µm ** *p* < 0.01; 155 µm * *p* < 0.05; 163 µm ** *p* < 0.01; 171 µm * *p* < 0.05); basal dendrites F(1, 608) = 0.005, n.s.

An analysis of CA1 pyramidal neuron morphology (Figure 5A) pointed out some intergroup differences. Even though measurements of basal (CTRL = 2434.6 ± 248.7 µm; (d)CASQ-null = 2393.7 ± 308.6 µm; unpaired Student’s t-test: *p* = 0.92) and apical dendritic length (CTRL = 4407.1 ± 295.1 µm; (d)CASQ-null = 3335.6 ± 673.7 µm; unpaired Student’s t-test: *p* = 0.1) showed no significant difference between the CTRL and (d)CASQ-null mice, a Sholl analysis revealed that the number of apical dendritic crossings along the Sholl rings was different in the CA1 pyramidal cells of the two groups (Figure 5B), being significantly reduced in (d)CASQ-null neurons.

### 2.4. Spatial Learning Evaluation

Considering the enhancement in synaptic plasticity and its role in the memory process, spatial learning was evaluated by using the paradigm of Morris Water Maze, a hippocampus-dependent learning test commonly adopted to assess long-term memory [34]. Before starting with training, a pre-training session was carried out and the swimming speed of mice was assessed, resulting in no difference between the groups (two-way ANOVA test: F(1, 8) = 0.8953, *p* = 0.3719). Throughout the training sessions, the (d)CASQ-null mice did not show any motor impairment; in particular, the ability to swim and the resistance in swimming seemed not to be affected by the ablation of CASQ.

**Figure 6:**
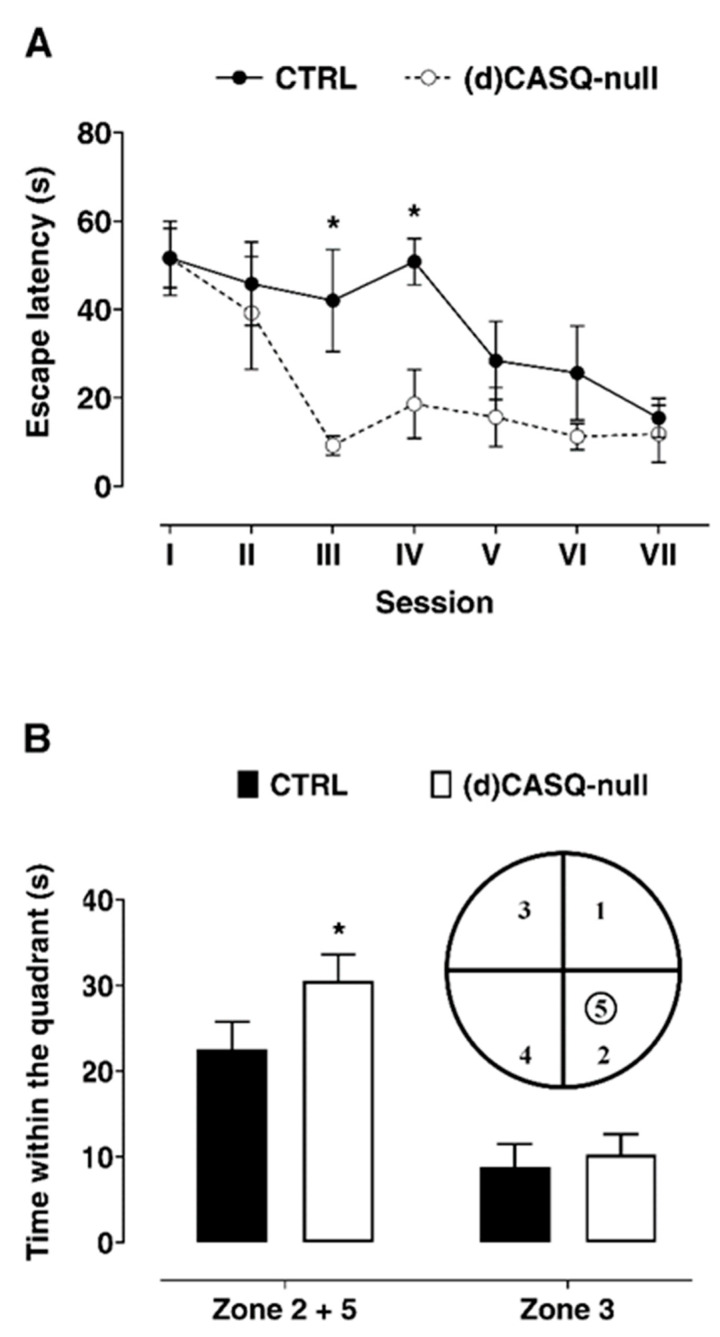
Evaluation of spatial learning using the Morris Water Maze paradigm. (**A**) Latency to reach the platform calculated considering the first trial of every session; each point represents the mean value (±S.E.M.) of all animals used. Two-way ANOVA test: session F(6, 68) = 5.7, *p* < 0.01; between animal groups F(1, 8) = 13.67 *p* < 0.01; Sidak’s multiple comparisons test * *p* < 0.05. (**B**) Probe test performed one day after the end of the training evaluated as the permanence time of the animal in the platform quadrant. Two-way ANOVA test: zone F(1, 8) = 20.06 *p* < 0.01; between animal groups F(1, 8) = 11.73, *p* < 0.01; Sidak’s multiple comparisons test * *p* < 0.05. *n* = 5 animals in both the CTRL and (d)CASQ-null mice.

In both groups of mice, as an effect of the learning process, the escape latency became progressively shorter, showing an asymptotic trend (Figure 6A). This finding was also validated by the probe test, revealing a preference for the quadrant in which platform was previously located (Figure 6B). Interestingly, the (d)CASQ-null mice performed significantly better than the CTRL in this task. Indeed, evaluating the performances in the first trial of every session, as an index of long-lasting memory, significant intergroup differences appeared on the third and fourth sessions of the training regarding escape latency (Figure 6A). This behavior in task performing resulted in much more time spent by the (d)CASQ-null mice in the goal quadrant compared to the opposite one (Figure 6B), indicating a shorter time needed by the (d)CASQ-null mice to develop a precise spatial preference for the goal.

## 3. Discussion

The present work aimed to gain insight into the role of CASQ in hippocampal activity. In this regard, although it was demonstrated that CASQ is expressed in the hippocampus [16], an investigation shedding light on CASQ involvement in synaptic plasticity and cognitive function was still missing. To achieve this purpose, we adopted a well-characterized CASQ knockout model, albeit used until now to study skeletal muscle function [22]. The main findings of the present work are: (i) CASQ2 is the isoform expressed in the hippocampus; (ii) Ca^2+^ dynamics are affected by a lack of CASQ; (iii) LTP in the Schaffer collateral-CA1 pathway is facilitated in mice lacking CASQ; and, finally, (iv) hippocampus-dependent spatial learning is improved in mutant mice.

It is well established that the induction of LTP entails Ca^2+^ signals at the pre- and postsynaptic levels; in particular, a transient postsynaptic elevation of intracellular Ca^2+^ concentration is required [35,36]. This Ca^2+^ increase is mediated by either the plasma membrane channels or InsP3 receptors and the RyR channels localized on internal stores, mostly ER [37]. In diverse synapse types, Ca^2+^ influx through *N*-methyl-D-aspartate (NMDA) receptors or voltage-gated Ca^2+^ channels [38,39] triggers the activation of RyRs. A role of RyRs in LTP responses has been demonstrated in different hippocampal regions [14,40], such as in the Schaffer collateral-CA1 synapses, where RyR channel inhibition prevents LTP induction or maintenance [41,42,43]. In contrast, lacking RyR3 results in the facilitation of LTP, induced by short tetanus stimulation, and in improving spatial learning in mutant mice [44].

While RyR involvement in plastic processes is widely supported, the role of proteins luminally controlling the RyR activity in neuronal plasticity is not known as yet. CASQ interaction with RyRs is well documented to be crucial in skeletal and cardiac muscle cell function, being an important regulator of sarcoplasmic reticulum Ca^2+^ release [45]. Changes in the association of CASQ with RyR channels amplify the direct effects of luminal Ca^2+^ concentration on the RyR activity [23,46,47,48].

The presence of CASQ in neurons, without any distinction between the two isoforms, has been demonstrated in the Purkinje neurons of the embryonic chicken cerebellum [49], and in the hippocampus of boars, where it is especially enriched [16]. However, it was not detected in rabbit whole-brain microsomal preparations [50], probably due to the expression being confined to discrete cerebral areas. In line with this evidence, our findings show the CASQ mRNA and protein expression levels, mostly CASQ2 isoform, in the hippocampus of CTRL mice, but not in (d)CASQ-null mice, where protein expression is abolished by the knockout construct. However, the preliminary results from the proteomic analysis do not point out any alterations in the protein expression pattern under CASQ deletion conditions, except for peroxiredoxin 6. This is a 25 kDa protein formed by 224 amino acids, showing both phospholipase A2 (PLA2) and GSH peroxidase activities; in addition, it also expresses a third enzymatic activity, lysophosphatidylcholine acyltransferase (LPCAT), that is coupled to its PLA2 activity [51]. Peroxiredoxin 6 in (d)CASQ-null mouse hippocampus exhibits in its primary structure a different amino acid residue in position 124 compared to control conditions. Nevertheless, this is a variant naturally occurrinh in the strain used to generate transgenic (d)CASQ-null animals, namely the C57BL/6J mice (UniProtKB source), which does not affect the peroxiredoxin 6 function.

From an electrophysiological point of view, our data highlight that under CASQ deletion conditions, the CA1 pyramidal neurons of mouse hippocampus exhibit peculiar electrophysiological characteristics. Indeed, hippocampal pyramidal neurons in (d)CASQ-null mice appear to be more excitable related to their depolarized RMP close to the AP threshold, differently from CTRL mice. On the other hand, the maximum AP frequency in CA1 neurons null for CASQ tends to be lower due to a significantly slower repolarizing phase of AP compared to control conditions. Importantly, the CA1 neurons in (d)CASQ-null mice show a significantly reduced peak amplitude of AHP, elicited following spike burst induction.

The AHP is referred to as a significant contributor to the regulation of neuronal excitability [33], and intracellular Ca^2+^ store is documented to play a role in AHP modulation [52]. In this view, Borde and coworkers [53] unveiled that the AHP shape may be affected by Ca^2+^ release from intracellular stores, and other authors have demonstrated that this Ca^2+^ source might contribute to AHP enhancement during aging [54,55,56]. In keeping with this evidence, the micromolar application of ryanodine reduces AHP by blocking Ca^2+^ release from the stores [53].

Ca^2+^-activated K^+^ channels may play a crucial role as possible mediators in coupling intracellular Ca^2+^ signals to membrane potential variations. Among these channels, it has been suggested that the BK type may stand for the link between the release of Ca^2+^ from intracellular stores and AHP, being voltage- and Ca^2+^-gated K^+^ channels [52,57]. The contribution of BK channels to action potential repolarization and AHP currents as well has been demonstrated [58,59,60]. In this scenario, we might hypothesize that the lack of CASQ may result in basal RyR Ca^2+^ leakage and that, when stimulated, Ca^2+^ release from the stores is reduced, inducing less activation of Ca^2+^-activated K^+^ channels and therefore a decrease in AHP amplitude. This speculation is supported by a large body of literature in the muscle of CASQ1-null mice, showing how the lack of CASQ promotes SR Ca^2+^ leak, elevated cytosolic Ca^2+^ levels, a reduction in SR storage and, finally, a reduced release during electrical stimulation or caffeine administration [23,47,48]. Additionally, two sets of data collected in the present work support this view: a) on one side, a trend of resting Ca^2+^ concentration toward higher values and a reduced endogenous buffer capacity estimated in (d)CASQ-null mice; and b) on the other side, a decreased Ca^2+^ transient amplitude in (d)CASQ-null mice when elicited by action potential trains.

Worthy of note is the fact that AHP was found to be inversely related to the LTP induction facility [52,54,61]. Indeed, evidence suggests that a relatively large AHP underlies much of the LTP impairment found in aged animals [62,63], and the pharmacological blockade of L-type Ca^2+^ channels, the inhibition of Ca^2+^ release from intracellular stores, or the manipulation of K^+^ channels enable the induction of LTP following a modest stimulation in these animals [52,61]. This is in line with a wealth of studies showing that AHP amplitude is involved in regulating the threshold for LTP induction [52,61,64,65,66,67,68,69,70,71].

Remarkably, LTP is a major reflection of synaptic plasticity, and it is the best-documented neuronal substrate for memory formation [72,73,74,75,76,77]. Over the decades, accumulating evidence has identified an essential role of the hippocampus for the acquisition and recall of spatial memories in mammals [78,79], and inhibitors of hippocampal LTP have been found to block spatial learning [80,81]. In line with these observations, we found that CASQ deletion in hippocampal neurons results in an enhanced LTP and improved spatial learning, evaluated by applying the paradigm of the Morris Water Maze.

Although further experiments will be needed to investigate the molecular mechanisms underlying the effects of CASQ deletion on hippocampal neurons and network function in depth, to our knowledge this is the first work that points to a role of a protein luminally controlling RyR activity (i.e., CASQ) in synaptic plasticity and cognitive processes. Even though the findings are unexpected, this is not the first work in which the lack of commonly occurring ER proteins results in LTP and spatial learning facilitation [44].

On the other hand, our findings also show that CASQ deletion affects the development of the dendritic tree, resulting in a minor complexity. This alteration was especially evident analyzing the apical dendritic trees of CA1 neurons, which extend in the stratum radiatum; consistently, the apical dendrite length tended to be on average less in comparison to the CTRL conditions. However, despite the reduction in dendrite branching, no significant difference was found in fEPSP, as shown by the input/output curves, indicating an unchanged basal synaptic transmission when Schaffer collaterals in the stratum radiatum were stimulated. Nevertheless, the relationship between the fiber volley amplitude and the stimulus intensity suggested that a lower number of presynaptic terminals were recruited in (d)CASQ-null mice to elicit a response similar to that of CTRLs. Consistently, the evoked synaptic responses (PSCs) recorded by whole cell patch clamp in CA1 neurons were not dissimilar in (d)CASQ-null mice compared to the controls. Finally, the synaptic facilitation induced by LTP was unaffected by this morphological modification. Explaining these findings is not trivial at all. It could be assumed that the contribution of each synapse to CA1 neurons in (d)CASQ-null mice increases to compensate for a possible reduction in presynaptic axons/branches. In turn, this could mean a rise of neurotransmitter released into the synapse or a postsynaptic mechanism compensating for the reduction in the presynaptic drive. This idea is perfectly consistent with the general hypothesis, known as “synaptic scaling”, according to which postsynaptic receptor density, in particular α-amino-3-hydroxy-5-methyl-4-isoxazolepropionic acid receptor (AMPAR), can be adjusted in order to homeostatically regulate the neuronal activity level, possibly by regulating the DNA methylation/demethylation ratio [82].

## 4. Closing Remarks

Together, our findings provide insights about the involvement of CASQ in the hippocampal neuron structure and function. Additionally, this study confirms the role of store-derived Ca^2+^ signals in synaptic plasticity and cognitive functions and suggests that the modulation of intra-ER sequestering proteins may affect these processes.

## 5. Materials and Methods

### 5.1. Double (d)CASQ-Null Mice

CASQ1-null and CASQ2-null mice were generated as previously described [21,27]. Briefly, (d)CASQ-null mice lacking both CASQ isoforms were generated by cross-breeding the pre-existing CASQ1-null and CASQ2-null mice [22]. C57BL/6J mice were used as wild-type controls (CTRL) (Charles River, Italy). All the mice were housed in micro-isolator cages, maintained at an ambient temperature of 22 ± 1°C, with a 12 h light and 12 h dark cycle (light on at 6 a.m. and off at 6 p.m.), and with free access to water and food. The mice were generated and bred at the University of Chieti-Pescara’s animal facility (Prog. 40/CEISA) and then were transferred to the facility of the University of Urbino before performing experiments.

### 5.2. Animal Care

The animal care and use were conducted in accord with the guidelines of the Ethics Committee of the University of Urbino Carlo Bo. All efforts were made to minimize the number of animals used. Adult male mice (CTRL *n* = 30; (d)CASQ-null *n* = 25) were killed to carry out brain explant; an overdose of sodium thiopental was used to sacrifice mice for biochemical evaluations, while ketamine anesthesia followed by decapitation was applied to kill the mice for electrophysiological experiments (according to D.lgs. 26/2014 Annex IV: Methods of killing animals).

### 5.3. Biochemical Assays

Hippocampi were quickly dissected from the brains of mice and stored at -80°C until use. The CASQ mRNA expression was evaluated. The extensor digitorum longus (EDL) muscles were also surgically removed for RNA extraction and used as a reference tissue. The CASQ protein expression was assessed by Western blot (WB), and preliminary experiments of proteomic analysis were also carried out.

#### 5.3.1. Gene Expression Analysis

Total RNA was extracted from the hippocampi and EDL muscles of CTRL mice (*n* = 3). The mRNA purification was performed using the RNeasy Mini kit (Qiagen, Milano, Italy) according to the manufacturer’s instructions and, finally, the contaminant DNA was digested with DNase I enzyme (Thermo Fisher Scientific, USA). The concentration of RNA was determined by measuring the absorbance using a (Varian Cary 50 Bio UV-Visible Spectrophotometer, Agilent Technologies, USA) spectrophotometer and 2.5 μg was reverse-transcribed with RT^2^ First Strand Kit for cDNA synthesis (Qiagen, Milano, Italy) to obtain cDNA. Real-time Polymerase Chain Reaction (RT-PCR) amplifications were conducted using RT^2^ SYBR Green Mastermixes (Qiagen, Italy) according to the manufacturer’s instructions, with 400 nM primers and one microliter of cDNA for a final reaction volume of 25 μL into each tube. Specific primers used for RT-PCR were: Casq1 (Casq1-F: 5′-CCTCCCAAGTCTCGTACATAC-3′ and Casq1-R: 5′-AGCCTATGACCATCCCAGA-3′); Casq2 (Casq2-F: 5′ CTCCATCCAGATACTGTCAGC-3′ and Casq2-R: 5′-TTGACCCAGATGACTTTCCAC-3′) (both from IDT, TEMA RICERCA, Italy) and Glyceraldehyde 3-phosphate dehydrogenase (GAPDH) (Qiagen, Italy). Thermocycling was conducted using a Rotor-Gene Q (Qiagen, Italy) initiated by a 10 min incubation at 95°C, followed by 40 cycles (95 °C for 15 s; 60 °C for 30 s), with a single fluorescent reading taken at the end of each cycle. Each reaction was conducted in triplicate and completed with a melt curve analysis to confirm the specificity of amplification and the lack of primer dimers. Quantification was performed according to the method described by Pfaffl [83], using the expression level of GAPDH as a reference.

#### 5.3.2. Western Blot (WB) Analysis

Hippocampi from CTRL and (d)CASQ-null mice (*n* = 3 for each group) were homogenized and samples were prepared as previously described [84]. Blots were incubated with the following primary antisera: anti-Calsequestrin, dilution 1:1000, rabbit polyclonal, PA1-913 (Thermo Fisher Scientific, USA); anti-Actin, dilution 1:400, rabbit polyclonal (Sigma-Aldrich, Milano, Italy). Then, the appropriate secondary antibodies conjugated with horseradish peroxidase, dilution 1:3000 (Bio-Rad, Italy), were applied. Immune complexes were visualized using the Clarity western ECL substrate chemiluminescent detection reagent (BioRad, Italy) following the manufacturer’s instructions, and the obtained autoradiographic films were quantified by ImageJ software (National Institutes of Health, USA) using the actin levels as a loading control.

#### 5.3.3. Proteomic Analysis

Hippocampi from CTRL and (d)CASQ-null mice (*n* = 3 for each group) were homogenized by pestle with lysis buffer containing 7M Urea, 2M Thiourea, 4% (w/v) CHAPS, 10 mM DTE, 20 mM Tris-base, 1 mM EDTA, 1 mM PMSF and Protease Inhibitor (one tablet in 2 mL of distilled water, 80 µl were added to 2 mL of lysis buffer) (Roche Applied Science, Germany). Following sonication, the lysates were centrifuged at 14,000 rpm for 30 min at 10 °C and the protein concentration was determined in the supernatant by a Bradford assay [85]. Two-dimensional electrophoresis (2-DE) was carried out as previously described [86]. For the analytical and semi-preparative 2DE, 100 µg and 400 µg of total protein, respectively, were resuspended in rehydration buffer containing 8M UREA, 4% (*w*/*v*) CHAPS, 65mM DTE, 0.8% IPG buffer (3–10), 0.6 % Bromophenol Blue and loaded onto 24 cm non-linear Immobiline strips, pH range 3-10 (GE Healthcare). The strips were focused onto IPGphor (GE-Healthcare) at 20 °C with the following gradient: 30 V for 10 h, 200 V for 1 h, 300 V for 30 min, from 300 V to 5000 V over 4 h and stabilized at 5000 V for 20 h. After isoelectrofocusing (IEF), strips were reduced, alkylated and then placed on 9–16% polyacrylamide linear gradient gels and 40 mA/gel constant current was applied.

Analytical gels were stained with silver nitrate [87], while semipreparative gels for mass spectrometry analysis were stained with Brilliant Blue R250. Gel images were acquired by the Fluor S-MAX multi-imaging system (BioRad, Italy), and the data were analyzed, including spot detection, quantification, and normalization, with the Image Master 2D Platinum version 5 software (GE Healthcare, Italy). Proteins differentially expressed from the CTRL and (d)CASQ-null mice were in gel digested with trypsin according to Shevchenko’s protocol [88], and an LC-ESI-MS/MS analysis was performed using a Q-TOF micro™ mass spectrometer (Micromass, UK) equipped with a Z-spray nanoflow electrospray ion source and a CapLC (Waters, USA), as previously described [89,90]. For protein identification, the MS/MS spectra were used as a query in MASCOT (Matrix Sciences, London, UK). The protein identity was assessed (in addition to MASCOT score) with at least three-peptide coverage and consistency with the pI/Mw inferred by 2D-PAGE.

### 5.4. Electrophysiogical Recordings

Brains from the CTRL and (d)CASQ-null mice (*n* = 16 and *n* = 14, respectively) were quickly removed and parasagittal, 400-micrometer-thick brain slices were obtained as previously described [91,92]. Field potential and whole-cell patch clamp recordings were carried out. Moreover, in a set of acute slices from the CTRL and (d)CASQ-null mice, experiments addressed to evaluate the endogenous buffer capacity, resting Ca^2+^ concentration, and calcium influx using fluorescence-based calcium imaging techniques were also performed. All the recordings started following a slice equilibration period in the recording chamber. Electrophysiological data analyses were carried out offline using the WinWCP software and WinFluor imaging software (John Dempster, Strathclyde University, UK) for Ca^2+^ imaging.

#### 5.4.1. Field Potential Recordings

The synaptic plasticity was evaluated by measuring (a) the basal synaptic transmission (input/output curves) and (b) the ability to elicit long-term potentiation (LTP) in the Schaffer collateral-CA1 pathway of mutant and CTRL mice, as previously described [91]. Input/output curves were obtained applying to the slice square pulses of current (300 μs in duration) with A385 stimulus isolator (World Precision Instruments, USA); fEPSPs slopes were measured in response to single electrical stimuli of increasing magnitude (from 0 to 140 µA, increments of 20 µA). The Schaffer collaterals were stimulated using a stimulus pattern including 10 trains of 100 Hz applied for 0.1 s separated by an interval of 0.2 s [84]; the fEPSP was then monitored by recordings for 40 min. The slopes (between 10% and 80% of max) of the fEPSP were analyzed and taken as measures of synaptic strength. The values were normalized to the mean value obtained over the last 20 min of the baseline period and expressed as a percent of this baseline value [84].

#### 5.4.2. Patch Clamp Recordings

Electrophysiological properties of CA1 pyramidal cells were investigated in mutant and CTRL mice using patch clamp recordings in whole-cell configuration carried out under visual guidance, as previously described [84,91]. The somata of CA1 pyramidal neurons to be recorded were identified based on their typical shape; the resting membrane potential (RMP), input resistance (IR), capacitance (C), and cell excitability were determined [91]. Action potentials (APs) were evoked by the somatic injection of increasing steps of depolarizing currents, and the amplitude and shape of the first elicited spike and the maximum firing frequency were evaluated.

Afterhyperpolarization (AHP) was evoked by delivering depolarizing current pulses (1 s) every 60 s through the patch pipette to elicit a sodium spike bursts of approximatively 20 action potentials; the AHPs in CTRL and mutant conditions were induced at the same membrane potential by clamping it with DC current injection. The post-burst AHP was measured as the difference between the pre-pulse membrane voltage and the most hyperpolarized membrane potential reached after the offset of the depolarizing current.

Finally, postsynaptic currents (PSCs) were evoked as previously described [84].

Recordings were discarded if the initial series resistance was >30 MΩ, if the series resistance measured at the end of the experiment had changed (±5MΩ), or if DC offset exceeded 5 mV after withdrawal from the cell.

#### 5.4.3. Calcium (Ca^2+^) Imaging

Ca^2+^ imaging recordings were carried as previously described [93,94]. Briefly, the experiments were carried out using a Zeiss Axioskop microscope (Carl Zeiss International, Milan, Italy) equipped with a 40× water immersion objective and the Orca Flash 4.0 CCD camera (C11440, Hamamatsu, Japan), an Axopatch-200B amplifier (Axon Instruments, San Jose, CA, USA), and WinFluor software (Strathclyde Imaging Software V 3.8.7, John Dempster, University of Strathclyde, UK). Patch pipettes were filled with an intracellular solution containing, in millimolar: 100 potassium gluconate, 26 KCl, 8 NaCl, 0.2 EGTA, 10 HEPES, 3 Mg_2_ATP, 0.3 GTP, and 100 µM cell impermeant Fluo-4 Pentapotassium Salt (Life Technologies, Italy) (pH = 7.2; 290 mOsm). Fluorescence images (200 × 200 pixels) were acquired at a 100 Hz frequency using an FITC excitation filter of 450–490 nm, and the fluorescence values were expressed as ΔF/F_0_, where F_0_ is the fluorescence at resting conditions. The fluorescence evaluation started immediately upon break-in, and, during the loading phase, the F_0_ and the Ca^2+^ transient in response to a single action potential were monitored. The steady state was reached after about 15–20 min. Moreover, once the cells were fully loaded with Fluo-4, the fluorescence changes in response to high frequency stimulations (20–100 Hz, 600 ms) were evaluated.

In each recorded neuron, the endogenous buffer capacity (κ_S_), unperturbed Ca^2+^ transient, and intracellular Ca^2+^ concentration at resting condition [Ca^2+^]_0_ and during a single action potential Δ[Ca^2+^]_AP_ were calculated according to Maravall and collegues [95], as previously described [93].

### 5.5. Morphological Analysis

Throughout the whole-cell recordings, except those for Ca^2+^ imaging, biocytin was injected into CA1 neurons by patch pipette. Following slice fixation with paraformaldehyde (4% PFA in phosphate buffer saline (PBS); Sigma-Aldrich, Italy), biocytin was detected as previously described [84,91]. A confocal microscope (Leica TCS-SL), equipped with Argon and He/Ne laser sources, was used to perform morphological reconstruction of each labelled pyramidal cell. Reconstructed neurons without clear dendritic cutting at the slice surface were considered for morphological analysis. The total length of pyramidal cell dendrites was measured using the NeuronJ software [96], whereas a Sholl concentric ring analysis was applied to assess the dendrite tree complexity, as previously described [84].

### 5.6. Morris Water Maze

Adult CTRL and (d)CASQ-null male mice (*n* = 5 for each group) were tested for spatial learning using the paradigm of Morris water maze, as previously described [84]. A pre-training session without a platform was carried out to accustom the mice to water. Afterwards, the training protocol was administered, consisting of two sessions per day (8 a.m. and 2 p.m.) of four trials each (60 s with an intertrial time of 60 s) over 3 consecutive days. A video tracking system Smart-BS (2biological Instruments, Italy) was used to record the time taken to reach the platform (escape latency) as an index of learning ability. Moreover, to obtain a more reliable measure of learning, the day after the end of the training the mice were tested for probe task by removing the platform from the pool and tracking the swim path for 60 s. To evaluate the result of this test, the maze was virtually divided into four quadrants, one of which was centered on the position formerly occupied by the platform (goal quadrant); the time spent and the distance swam in the goal quadrant as compared to those in the other ones were measured.

### 5.7. Statistical Analysis

Before the application of parametric statistics, each data distribution was explored using the Shapiro–Wilk test to assess the normality of the distributions. The data were expressed as mean ± SEM. Differences between the experimental groups were statistically evaluated using GraphPad Prism 8 Software. A series of unpaired Student’s t-tests and two-way ANOVA tests followed by Sidak post-hoc tests were appropriately applied to interpret the results. The significant difference was established at * *p* ≤ 0.05 and ** *p* ≤ 0.01.

## Figures and Tables

**Table 1:** Electrophysiological properties of CA1 neurons in CTRL and (d)CASQ-null mice.

	CTRL	(d)CASQ-Null
RMP (mV) **	−65.1 ± 2.3	−55.6 ± 2.6
IR MΩ	210.4 ± 19.1	249.7± 21.4
C pF	104.5 ± 9.1	93.6 ± 8.9
AP-T (mV) **	−33.2 ± 2.2	−41.0 ± 1.2
AP-A (mV)	101.7 ± 3.6	101.8 ± 4.2

RMP= resting membrane potential (CTRL *n* = 23 cells from 6 animals, (d)CASQ-null *n* = 20 cells from 6 animals); IR = input resistance (CTRL *n* = 23 cells from 6 animals, (d)CASQ-null *n* = 20 cells from 6 animals); C= capacitance (CTRL *n* = 23 cells from 6 animals, (d)CASQ-null *n* = 20 cells from 6 animals); AP-T = action potential threshold (CTRL *n* = 23 cells from 6 animals, (d)CASQ-null *n* = 19 cells from 6 animals); AP-A = action potential amplitude (CTRL *n* = 23 cells from 6 animals, (d)CASQ-null *n* = 19 cells from 6 animals). Student’s t-test ** *p* < 0.01.

**Table 2:** Calcium parameters recorded in CA1 pyramidal neurons in CTRL and dCASQ-null mice.

	CTRL	(d)CASQ-Null
[Ca^2+^]_0_ (nM)	39 ± 6.5	47 ± 18.9
Δ[Ca^2+^]_AP_ (nM)	12 ± 1.2	14 ± 2.8
Unperturbed [Ca^2+^] (nM)	40 ± 12.8	61 ± 21.4

[Ca^2+^]_0_ = resting calcium concentration (CTRL *n* = 13 cells from 4 animals, (d)CASQ-null *n* = 10 cells from 3 animals); Δ[Ca^2+^]_AP_ = calcium transient evoked by single action potential (CTRL *n* = 13 cells from 4 animals, (d)CASQ-null *n* = 10 cells from 3 animals); unperturbed [Ca^2+^] = calcium transient evoked by single action potential in the absence of exogenous calcium buffer (CTRL *n* = 7 cells from 4 animals, (d)CASQ-null *n* = 6 cells from 3 animals).

## Abbreviations

AP	action potential
AHP	afterhyperpolarization
C	capacitance
CASQ	Calsequestrin
CASQ1 and CASQ2	CASQ type-1 and type-2 isoforms
CICR	Ca^2+^-induced Ca^2+^ release
CR	calreticulin
ER	endoplasmic reticulum
fEPSP	field excitatory postsynaptic potential
HFS	high frequency stimulations
IR	input resistance
LTP	long-term potentiation
RMP	resting membrane potential
RyR	ryanodine receptor.
